# Nutrition and Lifestyle Coaching: An Interprofessional Course for Pharmacy, Medical, and Dietetic Students

**DOI:** 10.7759/cureus.48302

**Published:** 2023-11-05

**Authors:** Farid G Khalafalla, Kelly K Eichmann, Anne VanGarsse, William Ofstad

**Affiliations:** 1 Preclinical Basic Sciences, Touro University California, College of Education and Health Sciences, Vallejo, USA; 2 Career and Technical Education, Clovis Unified School District, Clovis, USA; 3 Pediatrics, University of California Riverside, School of Medicine, Riverside, USA; 4 Pharmacy Practice, West Coast University, School of Pharmacy, Los Angeles, USA

**Keywords:** underserved communities, healthcare professional students, team-based learning, interprofessional education, nutrition and lifestyle coaching

## Abstract

Background

Poor nutrition and lifestyle choices are major contributors to the development and progression of various chronic diseases. Enhancing patients’ awareness of healthy nutrition and lifestyle habits by interprofessional healthcare teams can play a significant role in tackling many chronic diseases, particularly in underserved communities with inequitable access to healthcare and educational opportunities. However, healthcare professionals are not adequately prepared to provide effective, culturally competent nutrition and lifestyle coaching due to a lack of emphasis on these topics in the curricula of many healthcare professional programs.

Objective

This study introduces a virtual, interprofessional, team-based elective course to address the curricular gap in nutrition education among healthcare professional programs.

Methods

Quantitative and qualitative pre-/post-surveys were utilized to evaluate course impact on student’s knowledge, confidence in coaching families, and interprofessional competencies. Quantitative pre-/post-training scores were analyzed by a two-tailed, Mann-Whitney test, where P < 0.05 indicated a significant difference. Additionally, student learning outcomes were assessed using readiness assurance tests and application exercises, along with end-of-course presentations and mock interviews.

Results

Pre-/post-course assessments (n = 16) demonstrated significant improvement in students’ confidence in coaching families (20%; P = 0.01) and knowledge of coaching curriculum (87%; P < 0.001). Improvement in different interprofessional competencies ranged between 15% (P = 0.002) and 46% (P < 0.001). Course material/activities, facilitation, impact on learning new knowledge/skills/mindset, and application in future practice were applauded by 81-94% of students.

Conclusion

Positive outcomes of this course encourage future offerings and systematic incorporation of similar training in healthcare professional programs to prepare clinicians capable of transforming lives through interprofessional, patient-centered nutrition and lifestyle coaching.

## Introduction

Poor diet, lack of physical activity, and excessive weight gain are costly public health issues. These are linked to the onset of many chronic diseases [1] and result in an expected decrease in longevity [2]. In 2020 - 2021, life expectancy at birth in the United States has shown the biggest two-year decline since 1921 - 1923 [3]. These factors are implicated in the development and progression of a variety of chronic diseases, including heart disease, cancer, stroke, and diabetes mellitus, which become more prominent in underserved communities with inequitable access to healthcare. Poor lifestyle-related chronic diseases represent a major burden on the United States healthcare system and account for a significant percentage of annual deaths [3,4].

Lifestyle-related diseases can be effectively reduced through diet modifications, increased regular physical activity, and other positive lifestyle changes, such as smoking cessation and managing stress [5-7]. Healthcare professional teams can play a significant role in increasing patient awareness and assisting in the initiation of healthful lifestyle choices. This includes promoting the practice of following a healthy diet and incorporating physical activity as part of positive lifestyle choices that lead to health benefits, especially in underserved communities that generally demonstrate lower levels of health literacy and fewer nutrition and lifestyle educational opportunities. Hence, it is crucial to prepare the upcoming generation of healthcare professionals, so they are adequately trained on the basics of nutrition, healthy food, and lifestyle choices, along with mastering the interprofessional, culturally competent, patient-centered delivery of these concepts to inspire positive behavioral change and achieve effective preventive care. This necessitates placing special emphasis on interprofessional nutrition and lifestyle coaching in healthcare professional programs’ curricula. Currently, most healthcare professional programs do not incorporate meaningful, in-depth nutrition and healthy lifestyle education [8]. Nutrition and lifestyle education is either not sufficiently addressed or scantly embedded into other required courses without adequate training of healthcare students on real-life clinical application and healthy lifestyle coaching tips.

To address this curricular gap, an interprofessional team of educators in the arenas of medicine, dietetics, pharmacy, and cardiovascular research designed an interprofessional elective course focused on nutrition and lifestyle coaching for health professions students. The course aims to equip future frontline healthcare professionals with the foundational knowledge and skills to promote healthy lifestyle choices. This course was based on our previously published nutrition and lifestyle coaching program [9], with the inclusion of more comprehensive assessment tools. The elective course was facilitated in an interprofessional, team-based learning (TBL) format and included a series of nutrition and lifestyle coaching modules with interactive real-life application exercises. This interprofessional TBL instructional approach allowed medical, pharmacy, and dietetic students to learn with, from, and about one another in an active learning environment that enhances the attainment of Interprofessional Education Collaborative (IPEC) core competencies [10] i.e., ethics and values, communication, teamwork, and defining roles and responsibilities of each healthcare profession in educating patients about healthy nutrition and lifestyles [11]. IPEC core competencies and overall learning outcomes were in alignment with interprofessional education (IPE)-related accreditation standards for osteopathic medicine [Commission on Osteopathic College Accreditation (COCA)], pharmacy [Accreditation Council for Pharmacy Education (ACPE)], and dietetic [Accreditation Council for Education in Nutrition and Dietetics (ACEND®)] programs.

## Materials and methods

A total of 25 students participated partially or fully in the interprofessional team-based learning (TBL) elective course: 13 pharmacy students from California Health Sciences University, seven osteopathic medical students from Kansas City University and A.T. Still University, and five dietetic students from California State University, Fresno. Sixteen students completed post-course assessments.

The one-credit elective course comprised of eight, two-hour classes, which were held virtually via Zoom due to COVID-19 pandemic restrictions. The first session covered an overview of the causes of obesity and methods of adiposity assessment. The second session detailed the role of nutrition and lifestyle choices in cardiovascular diseases. The third and fourth sessions outlined considerations in culturally competent coaching and motivational interviewing. The fifth and sixth sessions covered the application of the 5-2-1-0 nutrition and lifestyle coaching curriculum that is founded on the promotion of consuming five servings of fruits/vegetables, spending two hours or less of recreational screen time, engaging in at least one hour of physical activity, and avoiding sugary beverages. Students were also trained on the utility of the 15-minute obesity prevention protocol published in the pediatric obesity clinical decision support chart, second edition, by the American Academy of Pediatrics. As part of the course assessments, students were invited to individually prepare an audio-recorded presentation that summarizes key principles and strategies related to nutrition and lifestyle coaching and their application in solving a patient case following the pharmacists' patient care process (PPCP). PPCP provides a stepwise approach that starts with collecting patient information pertinent to medical history and current clinical condition, assessing collected information for appropriate diagnosis and recommendations for optimal care, developing a personalized patient-centered healthcare plan, collaborative implementation of the plan with the patient and other healthcare professional members of the team, and finally monitoring and evaluating the effectiveness of the care plan and suggesting modifications, if needed (PPCP steps adopted from Joint Commission of Pharmacy Practitioners, 2014). In addition, students were given the opportunity to apply the acquired coaching and motivational interviewing skills by running a mock interview following the pediatric obesity clinical decision support chart 15-minute obesity prevention protocol. The final two sessions of the course included instructor feedback on students’ recorded presentations and mock interviews. The course concluded with a student representative from each discipline (i.e., pharmacy, osteopathic medical, and dietetic programs) presenting a summary of the knowledge, skills, and behaviors acquired throughout the elective course.

The interprofessional TBL elective course included pre-class readiness material, individual readiness assurance tests (iRATs) and team readiness assurance tests (tRATs) at the beginning of each class, instructor-led debriefing on RATs guided by a PowerPoint presentation, and 4S-designed team application exercises founded on the following constructs: significant problem, same problem, specific choice, and simultaneous reporting. The tRATs and team application exercises enabled students from interdisciplinary healthcare professional programs to learn with, from, and about one another’s ethical values, roles and responsibilities, and to practice professional, effective communication among an interprofessional team, in alignment with IPEC core competencies.

Students’ learning outcomes were assessed using iRATS, tRATs, and team application exercises in each class, along with an end-of-course audio-recorded presentation that summarizes key foundational concepts learned from the course and their application in a patient case, and a mock interview to demonstrate coaching and motivational interviewing skills using a 15-minute obesity prevention protocol. In addition, students’ progress was evaluated by a mixed methods approach using both quantitative and qualitative surveys. The quantitative pre-/post-surveys assessed progress in students' confidence in mentoring families, knowledge of the 5-2-1-0 curriculum, awareness of healthy nutrition, daily dietary habits, and interprofessional competencies. The qualitative end-of-course survey enabled students to reflect on course strengths, areas for improvement, and insights.

**Figure 1:**
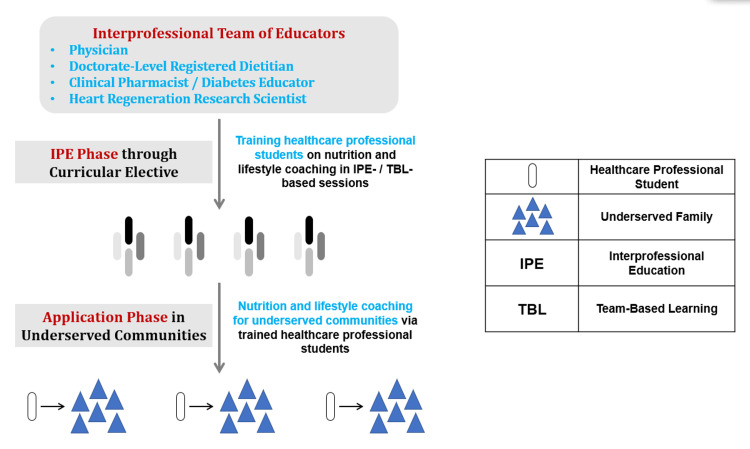
Overview of the IPE Nutrition and Lifestyle Coaching Platform and Volunteer Real-Life Opportunities to Coach Underserved Communities. A schematic outlining the interprofessional educational (IPE) framework for training healthcare professional students i.e., pharmacy, dietetic, and medical students, on culturally competent nutrition and lifestyle coaching for underserved communities. This IPE framework is facilitated by an interprofessional team of educators to ensure a quality learning environment that addresses the discussed topics holistically from clinical, research, and community perspectives. Additionally, the schematic demonstrates the voluntary follow-up real-life opportunities for trained students to apply the acquired skills through coaching the underserved Hispanic community in the Calwa neighborhood, Fresno, California.

Following course completion, students were offered the opportunity to apply learned skills by providing nutrition and lifestyle coaching for underserved community members in the Calwa neighborhood, located in Southeast Fresno County in California (Figure 1).

Quantitative pre-/post-survey scores are demonstrated as the means ± SE following statistical analysis using the two-tailed, Mann-Whitney test, where P < 0.05 indicated a statistically significant difference. Statistical analysis was performed using GraphPad Prism Software, version 9.4.1.

This study was approved by the institutional review board (IRB) at Rocky Vista University under an Expedited category (RVU IRB# 2019-0019).

## Results

**Figure 2:**
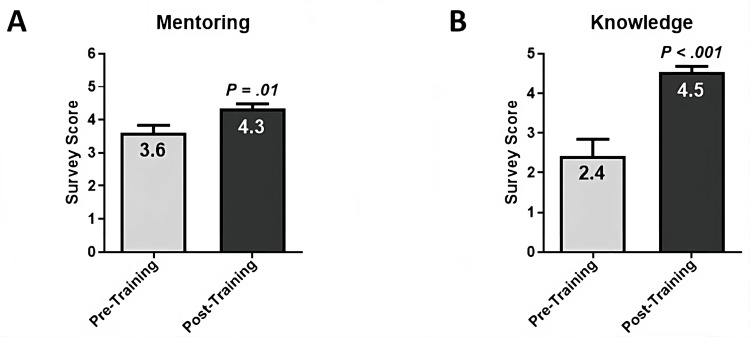
Pre-/Post-Course Assessment of Students’ Confidence in Family Mentoring and Knowledge of Coaching Curriculum. (A) The Mentorship domain of the survey comprised three questions on students’ confidence about answering questions on obesity, preparedness to discuss with families obesity prevalence and the importance of living healthy lifestyles, and preparedness to coach families on healthy lifestyle choices. Post-course students’ scores demonstrated a 20% increase (from 3.61 ± 0.23 to 4.33 ± 0.16, P = 0.01). (B) The Knowledge domain of the survey comprised one question on students’ understanding of the 5-2-1-0 nutrition and lifestyle coaching curriculum and how to set a goal with families. Post-course students’ scores demonstrated an 87% increase (from 2.43 ± 0.42 to 4.54 ± 0.14, P < 0.001). Statistical analysis was performed using a two-tailed, Mann-Whitney test, where P < 0.05 indicated a statistically significant difference.

Pre-/post-course surveys were administered to evaluate the impact of the elective course on students’ confidence in handling questions related to obesity, preparedness to facilitate coaching sessions on healthy lifestyle choices, knowledge of the 5-2-1-0 curriculum, and its utility in goal setting for coached families. Students’ responses were scored using a 1 to 5 Likert scale with the following metrics: 1 = Strongly Disagree, 2 = Disagree, 3 = Neutral, 4 = Agree, and 5 = Strongly Agree. Compared to pre-course scores, post-course responses demonstrated a 20% increase in students’ preparedness for mentoring/coaching families (from 3.61 ± 0.23 to 4.33 ± 0.16, P = 0.01) (Figure 2A) and an 87% increase in students' knowledge of the 5-2-1-0 curriculum (from 2.43 ± 0.42 to 4.54 ± 0.14, P < 0.001) (Figure 2B).

**Figure 3:**
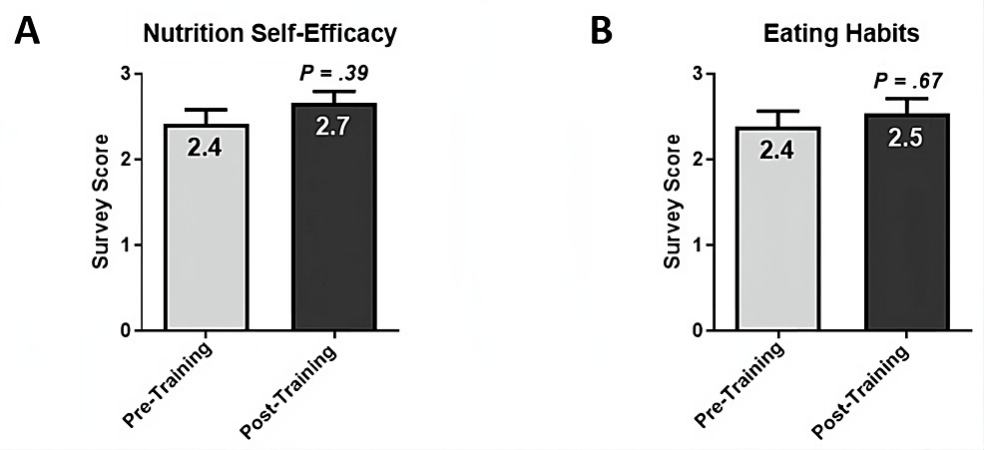
Assessment of Students' Progress in Nutrition Self-Efficacy and Dietary Habits using Nutrition Perception Screening Questionnaire 9 (NPSQ9). (A) The Nutrition Self-Efficacy domain of the questionnaire comprised five questions on the ability to manage to stick to healthy foods despite different barriers that may be encountered. Post-course students’ scores demonstrated an increasing trend of 10% (from 2.41 ± 0.17 to 2.66 ± 0.13, P = 0.39). (B) The Eating Habits domain of the questionnaire comprised four questions on the frequency of eating healthy foods and the thinking process to decide on healthy eating. Post-course students’ scores demonstrated an increasing trend of 6% in eating habits (from 2.38 ± 0.19 to 2.53 ± 0.18, P = 0.67). Statistical analysis was performed using a two-tailed, Mann-Whitney test, where P < 0.05 indicated a statistically significant difference.

To evaluate the impact of the elective course on nutrition self-efficacy and regular eating habits, students were invited to fill out a nine-question Nutrition Perception Screening Questionnaire (NPSQ9) [12] before and after the course. NPSQ9 posed five questions that reflect the ability to remain steady on healthy foods and four questions that evaluated dietary habits, including the rate of selecting healthy food options and substituting meals with snacks. Post-course students’ scores demonstrated increasing trends by 10% in nutrition self-efficacy (from 2.41 ± 0.17 to 2.66 ± 0.13, P = 0.39) (Figure 3A) and by 6% in eating habits (from 2.38 ± 0.19 to 2.53 ± 0.18, P = 0.67) (Figure 3B), but neither improving trends were statistically significant.

**Figure 4:**
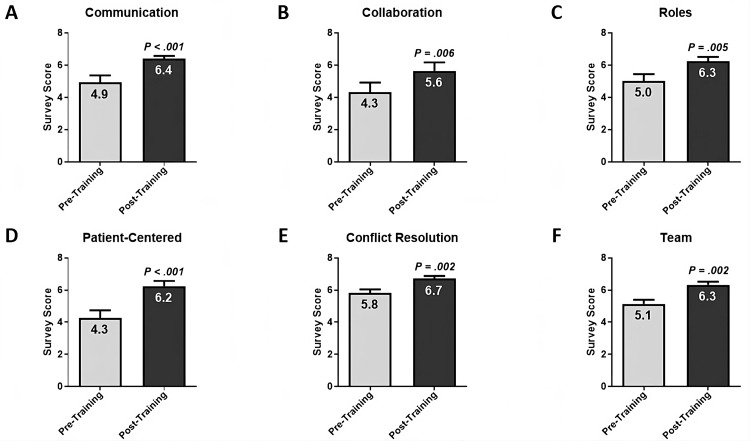
Assessment of Students' Progress in Interprofessional Competencies using Pre-/Post- Six-Domain Interprofessional Collaborative Competencies Attainment Survey (ICCAS). (A) The Communication domain comprised five questions. Post-course students’ scores demonstrated a 30% increase (from 4.92 ± 0.44 to 6.40 ± 0.17, P < 0.001). (B) The Collaboration domain comprised three questions. Post-course students’ scores demonstrated a 30% increase (from 4.33 ± 0.59 to 5.65 ± 0.52, P = 0.006). (C) The Roles domain comprised four questions on comprehending the roles and responsibilities of different healthcare specialties in patient care. Post-course students’ scores demonstrated a 25% increase (from 5.00 ± 0.44 to 6.25 ± 0.26, P = 0.005). (D) The Patient-Centered domain comprised three questions on conducting patient-centered care for families. Post-course students’ scores demonstrated a 46% increase (from 4.27 ± 0.47 to 6.25 ± 0.33, P < 0.001). (E) The Conflict Resolution domain comprised three questions. Post-course students’ scores demonstrated a 15% increase (from 5.82 ± 0.22 to 6.71 ± 0.17, P = 0.002). (F) The Team domain comprised two questions on teamwork to develop an effective patient care plan with clear responsibilities within the overlapping scopes of practice among healthcare team members. Post-course students’ scores demonstrated a 23% increase (from 5.13 ± 0.26 to 6.33 ± 0.19, P = 0.002). Statistical analysis was performed using a two-tailed, Mann-Whitney test, where P < 0.05 indicated a statistically significant difference.

At the end of the course, improvement in students’ interprofessional learning outcomes, which are aligned to IPEC Core Competencies, was evaluated using a retrospective pre-post, six-domain Interprofessional Collaborative Competencies Attainment Survey (ICCAS) [13,14]. Student responses were scored using a 1 to 7 Likert scale with the following metrics: 1 = Strongly Disagree; 2 = Moderately Disagree; 3 = Slightly Disagree; 4 = Neutral; 5 = Slightly Agree; 6 = Moderately Agree; 7 = Strongly Agree. ICCAS survey scores demonstrated statistically significant improvement in all of the assessed interprofessional learning outcomes: Communication scores increased by 30% (from 4.92 ± 0.44 to 6.40 ± 0.17, P < 0.001) (Figure 4A). Collaboration scores increased by 30% (from 4.33 ± 0.59 to 5.65 ± 0.52, P = 0.006) (Figure 4B). Roles and responsibilities scores increased by 25% (from 5.00 ± 0.44 to 6.25 ± 0.26, P = 0.005) (Figure 4C). Family-/Patient-centered approach scores increased by 46% (from 4.27 ± 0.47 to 6.25 ± 0.33, P < 0.001) (Figure 4D). Conflict resolution scores increased by 15% (from 5.82 ± 0.22 to 6.71 ± 0.17, P = 0.002) (Figure 4E). Finally, team functioning scores increased by 23% (from 5.13 ± 0.26 to 6.33 ± 0.19, P = 0.002) (Figure 4F).

Students’ satisfaction with the learning experience was assessed using a voluntary, anonymous post-course survey. Students’ responses showed that 63% of students completed all of the pre-class readiness material, whereas 37% completed only part of the pre-class readiness material. In addition, students’ responses demonstrated an overall satisfaction with the course design, material, and facilitation as indicated by the percentage of strongly agree/agree responses to the questions outlined in Table 1. Ninety-four percent (94%) of students applauded the course focus and learning outcomes and its relevance to their professional interests and development. Ninety-four percent (94%) of the students indicated that the design of the module activities is in alignment with the learning outcomes. Eighty-one percent (81%) of the students reported that the pre-class readiness material was beneficial. Ninety-four percent (94%) indicated that the scope covered during the session was appropriate for the allocated time. Eighty-eight percent (88%) of the students applauded the effective facilitation of the sessions and their engagement in active learning. Ninety-four (94%) of the students demonstrated that they overall enjoyed the sessions. Ninety-four percent (94%) of the students expressed that they have gained new knowledge, skills, or mindset. Ninety-four percent (94%) of the students demonstrated that they plan to use what they learned in the future. Detailed quantitative data of the post-course survey is presented in Table 1.

**Table 1:** Students’ Responses to Post-Course Feedback Survey.

	All		Some			None	
How much of the pre-class readiness material have you completed?	63%		37%			0%	
	Strongly Agree	Agree		Neutral	Disagree		Strongly Disagree
The focus and learning outcomes were relevant to my interests and development needs	63%	31%		0%	0%		6%
Activities were well-designed to meet the learning outcomes	50%	44%		0%	0%		6%
Pre-class readiness material were beneficial	50%	31%		13%	0%		6%
The scope covered was adequate for time allocated	63%	31%		0%	0%		6%
The facilitator(s) was (were) effective and engaged me in active learning	63%	25%		6%	6%		0%
Overall, I enjoyed this module	63%	31%		0%	0%		6%
I gained new knowledge, skills, or mindset from this module	75%	19%		0%	0%		6%
I plan to use what I learned here in the future	75%	19%		0%	0%		6%

Representative students’ comments on course strengths, areas for improvement, insights, and how the knowledge and skills from this course will be used in the future are outlined in Table 2.

**Table 2:** Representative Students' Comments from Post-Course Survey.

	Students’ Comments
What parts of this training do you expect to use in the future? Thinking of the soonest opportunity, describe where, when, and how.	* “Motivational Interviewing and giving information regarding food labels, calculation with BMI, and amounts of sugar in a product. The soonest opportunity will be with my extended family and as soon as Covid-19 is in control and its safe to do so, volunteering at community health fairs in Fresno county.”
	* “Learning how to effectively coach and speak to patients with empathy and understanding is definitely something I'm going to take with me to my IPPE and APPE rotations and retail internship. I now know how to better communicate with patients to gain their trust and show them empathy.”
	* “I plan on using this training at work when I counsel patients on lifestyle habits to go along with their pharmacologic therapy.”
	* “I really enjoyed the rule of 5-2-1-0, and I’m working on it. I already talked about it with some of my friends who are living out of country.”
Strengths	* “I really like the interprofessional collaboration and learning these topics from a different perspective. Also seeing the way that this course can be used in different parts of the medical field.”
	* “Breaking into groups and have them be a mix so everyone can see different point of views. Sharing real life examples is helpful in understanding current topics being taught.”
	* “Applications were beneficial in keeping us engaged and enhancing our learning.”
	* “The lectures are extremely helpful when learning the do's and don'ts of coaching, how to read nutrition labels, the importance of the 5-2-1-0 curriculum, and the transtheoretical model of change.”
	* “Leaving room for discussion and questions from the students, or real-life situations. It helps a lot.”
	* “I really enjoyed the readiness material, especially with links to supplemental articles and websites that are accessible for information. I also enjoyed the RATs, I think they allow for engagement with each other.”
	* “Very organized multiprocessors and very professional and knowledgeable.”
Areas for Improvement	* “More questions in the quizzes.”
	* “Virtually doing our mock interviews was tough and did not help me at all”
	* “I think for the mock interview videos, it would have been great to see an example from the professors even if it was a short video just to get a little guidance”
	* “Using technology to present a power-point presentation without any formal training in this application was the most stressful part of the program.”
	* “The applications could have been a little more challenging and thought-provoking to induce more discussion amongst students from different professional backgrounds.”
	* “When putting students in groups to discuss, maybe having someone who is in charge of starting conversation. This might be helpful in having longer discussions.”
	* “Maybe if instead of 2 hours was an hour and half would be great.”
Insights	* “I learned that the patient has more power than they know. I understood we help them understand that and help them be the ones to make the right changes in their lives.”
	* “I didn’t realize how much motivational interviewing and coaching skills were utilized in different fields of study.”
	* “I really liked seeing the non-pharmacy professors teach since they bring in different perspectives and expertise, where their lectures say so much good material.”
	* “I learned that I am not that great talking to patients and that I need to practice confidence in my speech and building a relationship with patients that I am coaching.”
	* “I enjoyed collaborating with other future professionals and learning about the 5-2-1-0 material. As a dietetic student I have still yet to come across that information. Also, learning how to convert grams of sugar to teaspoons was something I was unaware of.”
	* “I discovered that it is important to not just give information that’s helpful to our patients but being able to give that information out effectively is what is most important.”
Additional Comments	* “Even though this class was after 6 hours of lecture on a Monday, I enjoyed it.”
	* “I really enjoyed the time and the knowledge that I gained throughout this course.”

## Discussion

Acquiring basic nutrition and lifestyle knowledge and culturally competent coaching skills is crucial for all healthcare practitioners. Many future healthcare professional students are not adequately educated in this field or trained on how to effectively disseminate this information. The interprofessional, TBL elective course presented in this manuscript aimed to fill a knowledge and skills gap among students in training to become pharmacists, physicians, or registered dietitians. The focused content included nutrition basics and lifestyle factors associated with cardiovascular leading causes of death in the United States, along with culturally competent coaching techniques to effectively deliver the newly acquired knowledge to underserved communities. Students have successfully achieved the designed learning outcomes as demonstrated by various assessment methods, including individual- and team-based quizzes, clinical applications, presentations, and mock coaching interviews. The engaging interprofessional, TBL environment triggered intra- and inter-team discussions between healthcare professional students from different disciplines, which fostered a more profound understanding of the material from various perspectives. 

After completing the course, students were offered a volunteer opportunity to facilitate nutrition and lifestyle coaching sessions for a local underserved community in the Calwa neighborhood, Fresno, California. Calwa neighborhood is dominated by Hispanics (83.1 %) who are heavily involved in the farming industry. Calwa residents exhibit a rate of 12.74 emergency department visits for heart attacks per 10,000 individuals, placing Calwa in the 92nd percentile rank of cardiovascular disease prevalence nationally. Multiple students who were trained through our initial voluntary program [9] and the elective course presented in this manuscript were able to coach 58 underserved community members in Calwa. Coaching sessions were led by Spanish-speaking students to accommodate the linguistic needs of Calwa families. Coaching sessions were supported by visual brochures highlighting the 5-2-1-0 curriculum constructs at a literacy level that suits the community. Families were provided with educational brochures in Spanish in addition to grant-funded gift cards to promote healthy eating.

The influence of healthcare professionals who serve as community role models by practicing healthy nutrition and lifestyle habits can be far-reaching. While there was no post-course statistically significant increase in students’ nutrition self-efficacy and dietary habits, there was an upward trend indicating incremental progress. The lack of statistical significance could be due to the small number of participating students in this cohort or the need for longer-term follow-up as sustainable change of habits can take several months. Future course offerings will include a one-year follow-up to evaluate the sustainability of acquired knowledge, competencies, and healthful behavioral change over time. Increasing the sample size of participating students will be achieved by proposing the course inclusion in the core curriculum rather than offering it as an elective. The positive outcomes of the course and its value in equipping healthcare professional students with foundational skills for their future practice encourage us to propose its inclusion as a core course in alignment with interprofessional education accreditation standards of different health professions programs. This should ensure the exposure of all students to the essential nutrition and lifestyle coaching content presented in this course. Moreover, the interprofessional nature of the course will enable its offering to various healthcare professional programs locally, regionally, and nationally, to prove the generalizability of the course outcomes in different institutions.

Other nutrition and lifestyle training programs for healthcare professional students have been previously reported. This includes the Coaching, Health and Movement (CHAMPS) program [15], which was conducted only for medical students, and was further developed into our previously published voluntary interprofessional, team-based dietary coaching training program for pharmacy and dietetic students [9]. This voluntary program was then transformed into the elective course described herein with more developed assessment tools and increased diversity in participating healthcare professional student disciplines e.g., pharmacy, medical, and dietetic students. Offering this training opportunity in the form of an elective course is more appealing and rewarding to students, which increases chances of student exposure to these crucial culturally competent coaching skills and supports program sustainability to meet patient needs in underserved communities.

Overall, the elective course described in this manuscript provided healthcare professional students with foundational knowledge, skills, and practical tools to conduct nutrition and lifestyle coaching sessions for their patients, families, and communities. Students valued the interprofessional team discussions, clinical scenarios, course organization, and the resources provided in the course material. However, based on students’ feedback, there are multiple areas for improvement that will be taken into consideration in future course offerings. This includes increasing the number of iRAT questions, providing technology training to better prepare students for recorded presentations and mock interview assignments, using more challenging clinical application exercises, and during team application exercises, assigning a lead student to facilitate the discussion. The continuous quality improvement (CQI) process will be reinstituted in future course offerings by gathering student feedback through anonymous quantitative and qualitative surveys along with individual interviews or focus groups for more profound insights. In addition, to evaluate the course's effectiveness in preparing students for real-life nutrition and lifestyle coaching, feedback will be gathered from community members after coaching sessions by trained students. Finally, some refinements will be considered in the study design of future course offerings. A crossover design that provides a control student group will be pursued for a more rigorous comparative analysis of the course's effectiveness. Additionally, while this study was focused on evaluating student learning outcomes, future studies will assess the health outcomes of underserved community members coached by trained students, including changes in their nutrition self-efficacy and dietary/lifestyle habits.

The interprofessional training and community engagement framework presented herein can be adopted by various healthcare professional programs. Nonetheless, multiple aspects should be taken into consideration to ensure successful implementation. The logistics of organizing IPE activities represent a common challenge, which can be mitigated by proactive planning to identify mutually agreeable dates/times in the academic calendars of participating programs. Nutrition and lifestyle education material should be tailored to the health literacy level of the target community. Community members should be engaged in planning accessible healthy nutrition alternatives that fit their culture and financial capacity. It is also essential to meet community linguistic needs by involving student coaches fluent in the community’s spoken language.

## Conclusions

Nutrition and lifestyle education is an interdisciplinary topic that can be discussed from clinical, pharmacological, dietetic, and cultural perspectives. This overlap set the stage for the interprofessional team-based facilitation of our proposed elective course. The interprofessional, TBL platform helped healthcare professional students understand the significance of teamwork and effective interpersonal communication in providing enhanced patient care experience. In their future practice, trained students will be more open to work collaboratively with healthcare professionals from other specialties and rely upon one another’s knowledge and skills as true interdisciplinary team members towards increasing the quality of patient care. The course includes clinical application exercises and mock coaching interviews with direct feedback from a registered dietitian, which ensures students’ readiness to engage in real-life patient care.

## References

[1] Kirk S, Scott BJ, Daniels SR (2005). Pediatric obesity epidemic: treatment options. J Am Diet Assoc.

[2] Olshansky SJ, Passaro DJ, Hershow RC (2005). A potential decline in life expectancy in the United States in the 21st century. N Engl J Med.

[3] Arias E, Tejada-Vera B, Kochanek KD, Ahmad FB (2022). Provisional life expectancy estimates for 2021. NVSS.

[4] Murphy SL, Kochanek KD, Xu J, Arias E (2021). Mortality in the United States, 2020. NCHS.

[5] Eilat-Adar S, Sinai T, Yosefy C, Henkin Y (2013). Nutritional recommendations for cardiovascular disease prevention. Nutrients.

[6] Reidlinger DP, Darzi J, Hall WL, Seed PT, Chowienczyk PJ, Sanders TA (2015). How effective are current dietary guidelines for cardiovascular disease prevention in healthy middle-aged and older men and women? A randomized controlled trial. Am J Clin Nutr.

[7] Willett WC, Koplan JP (2006). Prevention of chronic disease by means of diet and lifestyle changes. Disease Control Priorities in Developing Countries 2nd edition.

[8] Crowley J, Ball L, Hiddink GJ (2019). Nutrition in medical education: a systematic review. The Lancet Planetary Health.

[9] Khalafalla FG, Covarrubias K, Fesperman M, Eichmann K, VanGarsse A, Ofstad W (2020). Enhancing nutrition and lifestyle education for healthcare professional students through an interprofessional, team-based training program. Curr Pharm Teach Learn.

[10] Collaborative IE (2016). Core competencies for interprofessional collaborative practice: 2016 update. Interprofessional Education Collaborative.

[11] Roh YS, Lee SJ, Mennenga H (2014). Factors influencing learner satisfaction with team-based learning among nursing students. Nurs Health Sci.

[12] San-Cristobal R, Navas-Carretero S, Celis-Morales C (2017). Capturing health and eating status through a nutritional perception screening questionnaire (NPSQ9) in a randomised internet-based personalised nutrition intervention: the Food4Me study. Int J Behav Nutr Phys Act.

[13] Archibald D, Trumpower D, MacDonald CJ (2014). Validation of the interprofessional collaborative competency attainment survey (ICCAS). J Interprof Care.

[14] Schmitz CC, Radosevich DM, Jardine P, MacDonald CJ, Trumpower D, Archibald D (2017). The interprofessional collaborative competency attainment survey (ICCAS): a replication validation study. J Interprof Care.

[15] Ronecker JC, Liu J, Newman RE, VanGarsse AM (2019). Coaching, health, and movement program (Champs) taught by medical students: a didactic curriculum and program analysis. Osteopathic Family Physician.

